# VEGFR-3 Signaling Regulates Triglyceride Retention and Absorption in the Intestine

**DOI:** 10.3389/fphys.2018.01783

**Published:** 2018-12-11

**Authors:** Trevor Shew, Nathan E. Wolins, Vincenza Cifarelli

**Affiliations:** Department of Medicine, Center for Human Nutrition, Washington University School of Medicine, St. Louis, MO, United States

**Keywords:** chylomicron, nitric oxide, lacteals, VEGFR-3, lipid trafficking, enterocytes

## Abstract

The lymphatic system transports dietary lipids absorbed and packaged as chylomicrons by enterocytes, for delivery to the bloodstream. Once considered a passive drainage, chylomicron entry into intestinal lymphatic vessels, or lacteals, is now emerging to be an active process controlled by a dynamic and complex regulation. Vascular endothelial growth factor (VEGF)-C, a major lymphangiogenic factor, regulates lacteal maintenance and function. Little is known about the role of its cognate tyrosine kinase VEGF receptor 3 (VEGFR-3) during lipid absorption. Here we investigated role of VEGFR-3 signaling in triglyceride (TG) absorption and distribution into tissues using the Chy mouse model, which bears an inactivating mutation in the tyrosine kinase domain of VEGFR-3 (heterozygous A3157T mutation resulting in I1053F substitution). Our data show that inactivation of VEGFR-3 tyrosine kinase motif leads to retention of TGs in the enterocytes of the small intestine, decreased postprandial levels of TGs in the plasma and increased excretion of free fatty acids (FFAs) and TGs into their stools. We further show that levels of nitric oxide (NO), required for chylomicron mobilization into the bloodstream, are significantly reduced in the Chy intestine after a fat bolus suggesting a critical role for VEGFR-3 signaling in the generation of NO during lipid absorption. Our data support the hypothesis that VEGFR-3 signaling plays an important role in chylomicron-TG entry into lacteals, possibly affecting TG trafficking to peripheral tissues.

## Introduction

Dietary long-chain fatty acids (FAs) and monoacylglycerols are absorbed on the apical membrane of enterocytes, repackaged into triglyceride (TG)-rich lipoproteins, or chylomicrons (CMs), and secreted into the intestinal lymphatic vessels, referred to as lacteals (Tso and Balint, 1986; Kohan et al., 2010; Mansbach and Siddiqi, 2010; Cifarelli and Abumrad, 2018). Recent reports have shown that chylomicron trafficking into lacteals is regulated by vascular endothelial growth factors (VEGFs), hormones, transcription factors and requires activation of specific signaling pathways (Van Dyck et al., 2007; Dixon, 2010; Choe et al., 2015; Bernier-Latmani and Petrova, 2017; Zhang et al., 2018) challenging the view of gut lymphatic endothelium being a passive barrier.

Defective growth of lymphatic vessels, or lymphangiogenesis, in the lacteals impairs lipid absorption and metabolism, as reported in several mice models of dysfunctional gut lymphatics due to genetic deletion of prospero homeobox gene Prox1 (Harvey et al., 2005), VEGF-C (Nurmi et al., 2015), Notch ligand delta-like ligand 4 (DLL4) (Bernier-Latmani et al., 2015), and adrenomedullin (Davis et al., 2017). VEGF-C, a major lymphangiogenic factor during embryonic development (Makinen et al., 2001; Karkkainen et al., 2004), regulates maintenance and function of intestinal lymphatics in adult mice (Nurmi et al., 2015) by binding its cognate tyrosine kinase VEGF receptor 3 (VEGFR-3), highly expressed in lymphatic endothelial cell (LECs). Upon binding with VEGF-C, VEGFR-3 forms homodimers (R3/R3) and undergoes intrinsic autophosphorylation on at least five cytoplasmic tyrosine residues (Dixelius et al., 2003). VEGFR-3 activation leads to protein kinase C-dependent activation of ERK1/2, implicated in cell proliferation (Pajusola et al., 1994; Fournier et al., 1995). VEGF-C can also bind to VEGFR-2, inducing formation of heterodimers between VEGFR-3 and VEGFR-2 (R3/R2) through a different autophosphorylation pattern (Dixelius et al., 2003; Nilsson et al., 2010). Postnatal deletion of *Vegfc* in mice leads to lacteal regression, defective lipid absorption and protects from diet-induced obesity and insulin resistance (Nurmi et al., 2015), without affecting any other lymphatic beds. Similar to *Vegfc* deletion, genetic ablation of *Vegfr3* in adult mice affects lacteal maintenance and function (Nurmi et al., 2015) although impact in lipid absorption was not investigated. Whether VEGFR-3 signaling affects TG absorption and distribution remains unclear. Here we investigate the role of VEGFR-3 signaling in TG trafficking from enterocyte into the circulation. We used the Chy mutant mouse, which carries an inactivating mutation in VEGFR-3 tyrosine kinase domain (heterozygous A3157T mutation resulting in I1053F substitution). Chy mutant mice present defective lymphatic vessels and develop lipid-rich chylous ascites at birth that resolves upon weaning (Karkkainen et al., 2001). To our knowledge, the mechanisms through which inactivation of VEGFR-3 signaling pathways affects lipid absorption and trafficking to blood circulation remains unknown.

## Materials and Methods

### Mice

All studies followed guidelines of the animal ethics committee of Washington University School of Medicine (St Louis, MO, United States). Chy mutant mice and littermate controls (gift from Dr. Gwendalyn Randolph, Washington University in St. Louis), all on the C57BL/6 background (Platt et al., 2013) were housed in a facility with a 12-h light-dark cycle and fed chow *ad libitum* (Purina, St Louis, MO, United States) or when indicated fasted for 12 h with *ad libitum* access to water.

### Triglyceride, Cholesterol, and Free Fatty Acid Measurements

Small intestines were collected at 120 min after intragastric administration of triolein (10 μL/g body weight) to 12-h fasted mice, divided into three equal parts (proximal, middle, and distal) and processed as previously described (Cifarelli et al., 2017). Total triglyceride, cholesterol, and free fatty acids were determined by homogenizing 50 mg of tissue in 2 ml of chloroform:methanol (2:1 v/v). Samples were centrifuged at 12,000 rpm for 10 min at 4°C. An aliquote, 50 μl, was evaporated in a 1.5 ml microcentrifuge tube. Triglyceride and cholesterol were determined by adding 100 μL of reagent to dried lipid extracts followed by incubation for 30 min at room temperature. Level of triglyceride and cholesterol in plasma was determined using Fisher Scientific kits (Fisher Scientific, PA, United States). Non-esterified fatty acid levels in plasma were determined using Wako kit (Wako Chemicals, Richmond, VA, United States) according to the manufacturer’s protocol.

### Immunohistochemistry

Small intestines were collected at 120 min after intragastric administration of triolein, opened longitudinally, divided into three equal parts (proximal, middle and distal), fixed in 10% formalin and embedded in paraffin. Cut sections (5 μm) were processed as previously described (Cifarelli et al., 2017). Sections were incubated in donkey serum (2%) and BSA (3%) for 1h at room temperature to block non-specific binding and then incubated overnight (4°C) with perilipin 3 primary antibody (Wolins et al., 2005) (1:1000), followed by fluorescently labeled (Alexa Fluor) secondary antibody (1:250). DAPI staining was used to identify nuclei. Images were taken using Nikon Eclipse TE2000-U microscope.

### Thin Layer Chromatography

Mice were monitored for stool excretion for 5h, continuously collected from 2 to 5 h of intragastric administration of triolein (10 μL/g body weight) and frozen until day of analysis. Stool samples were weighed, dispersed in PBS and lipids extracted as described previously (Schwartz and Wolins, 2007). The extracted lipids from 5 mg of feces were dissolved in hexane/ether (1:1) and loaded on Silica Gel HL chromatography plate (Analtech). The lipids were resolved in hexane/ether/acetic acid (70:30:1.2) and detected with iodine vapor as previously described (Skinner et al., 2009).

### Nitric Oxide Assay

Nitrite and nitrate levels were measured in small intestine using a fluorometric kit employing the Griess reaction (Cayman Chemical, Ann Harbor, MI, United States). After a 12-h fast, mice received intragastric triolein (10 μL/g body weight). Small intestines, collected at 120 min post gavage were gently washed in cold PBS, and snap frozen until samples (100 mg) were further processed following the manufacturer’s protocol.

### Statistical Analyses

Results are presented as means ± standard error (SE). Statistical significance is calculated using unpaired Student’s *t*-test. Statistically significant difference is defined as a *p*-value of ≤0.05.

## Results

### Inactivation of VEGFR-3 Signaling Decreases Plasma Triglyceride (TG) Levels After Lipid Bolus

**FIGURE 1:**
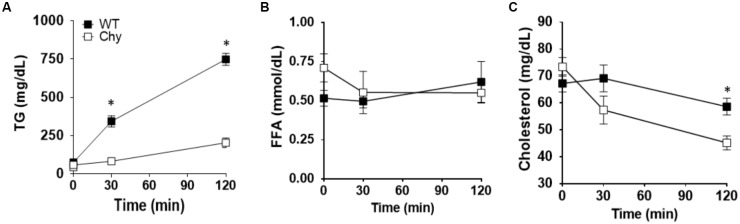
Chy mice have decreased TG and cholesterol level in plasma after a bolus of fat. An oral bolus of triolein (10 μl/g body weight) was administered to mice after an overnight fast. Plasma TG clearance was blocked by injection of Triton WR 1339, an inhibitor of lipoprotein lipase activity. Plasma was collected at the indicated times for TG **(A)**, FFA **(B)**, and cholesterol **(C)** measurements. Data are means ± SEM. ^∗^*p* < 0.05 by 2-tailed Student’s *t*-test.

Chy and control mice received an intra-gastric administration of triolein (10 μL/g body weight) after an overnight fast (12-h). Thirty minutes prior lipid bolus, both mouse groups received Triton WR 1339 by i.v. to block lipoprotein lipase activity (Otway and Robinson, 1967). Plasma TG levels were significantly decreased in Chy mice (83.7 ± 17 mg/dL) compared with control group (344.1 ± 35 mg/dL; *p* = 0.0006) at 30 min post gavage and remained lower at 120 min post gavage (203.9 ± 31 mg/dL as compared with 748.9 ± 78 mg/dL for Chy and control mice, respectively; *p* < 0.001) (Figure 1A). Level of FFAs in the plasma did not differ between the two mouse groups before and after intra-gastric administration of triolein (Figure 1B). The Chy mice exhibits decreased level of cholesterol in the plasma following intragastric administration of triolein, and this reached significance at 120 min post gavage (45.18 ± 2 mg/dL) compared with control group (58.64 ± 3.1 mg/dL; *p* = 0.029) (Figure 1C).

### TGs Both Accumulate in the Small Intestine and Is Excreted in the Feces in Chy Mice Following a Bolus of Lipids

**FIGURE 2:**
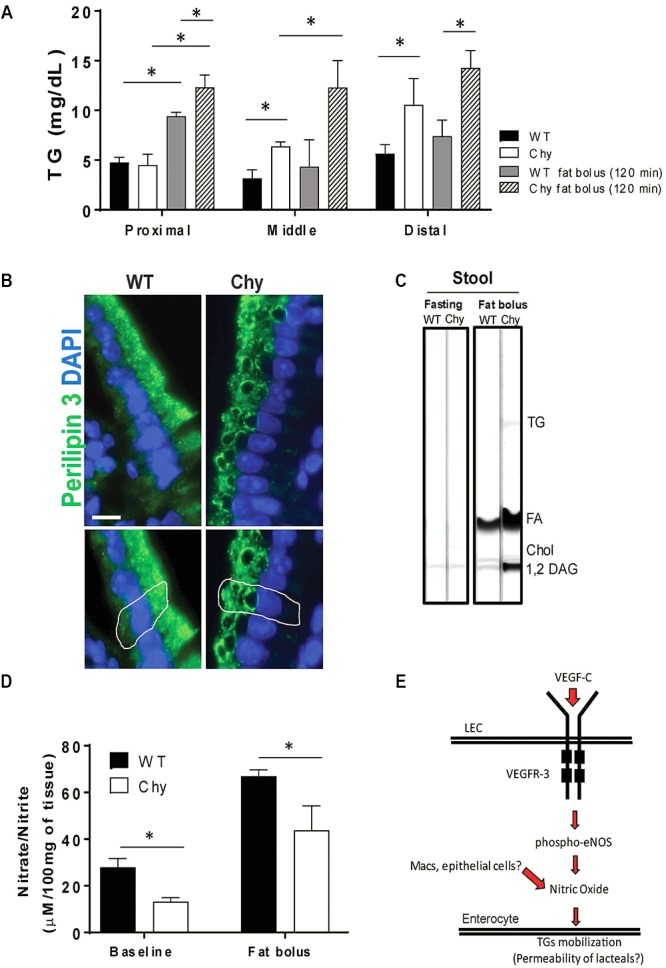
Chy mice accumulate TGs in the small intestine after a bolus of fat. Mice received an oral bolus of triolein (10 μl/g body weight) after overnight fasting (12-h). **(A)** Different sections of the intestine (proximal, middle, and distal) were collected from both mice groups at 120 min post gavage and analyzed for TG content. **(B)** Proximal small intestines were stained for Perilipin 3 (P3, green) a protein that coats lipid droplets (LD) and for DAPI (blue, nuclei). **(C)** Lipids were extracted from stool continuously for 5-h following an oral bolus of fat (10 μl/g of body weight) and analyzed by TLC. Stool from Chy mice show increased content of TG, FA and 1,2 DAG compared with stool from control mice. **(D)** Level of nitric oxide (nitrite/nitrate) measure by Griess reaction in intestine of Chy and littermate controls at baseline and after 120 min post lipid bolus. Scale bar is 30 μm. **(E)** Schematic model showing potential mechanism. Data are means ± SEM. ^∗^*p* < 0.05 by 2-tailed Student’s *t*-test.

We next examined intestine TG distribution in Chy mice before and after an intra-gastric bolus of triolein. In unchallenged conditions, TG accumulates in the small intestine of Chy mice with a significant shift toward the middle (6.3 ± 0.45 mg/dL vs. 3.1 ± 0.8 mg/dL in control group; *p* = 0.035) and distal sections (10.5 ± 2 mg/dL vs. 5.6 ± 0.9 mg/dL in control group; *p* = 0.007) (Figure 2A). Following intra-gastric administration of triolein, control mice show increased TG levels primarily in the proximal section (9.4 ± 0.4 mg/dL after bolus vs. 4.7 ± 0.5 mg/dL in unchallenged condition; *p* < 0.01) whereas the Chy mice present increased accumulation of TGs in the three different sections of the intestine (Figure 2A). Defective intestinal lipid processing in Chy mice is further shown by presence of larger cytoplasmic lipid droplets in enterocytes of the proximal small intestine, stained for Perilipin 3, compared with control mice (Figure 2B). In contrast, increased excretion of TGs, FAs and 1,2 Diacylglycerol (DAG) is observed in the feces of the Chy mice, collected at 5 h post gavage, compared with controls (Figure 2C).

Mobilization of postprandial chylomicrons from enterocytes into the circulation is initiated upon induction of nitric oxide (NO) signaling (Hsieh et al., 2015), although it is still not understood whether NO regulates chylomicron trafficking from enterocyte-to-lacteal or from lymph-to-blood. Chy mice intestine displayed lower levels of NO (nitrite/nitrate) measured at baseline (13.19 ± 1 μM/100 mg of tissue) as compared to WT mice (27.9 ± 2 μM/100 mg of tissue; *p* = 0.022), and after 120 min post fat bolus (30.09 ± 4 μM/100 mg of tissue) as compared with control mice (70.2 ± 3 μM/100 mg of tissue; *p* < 0.05) (Figure 2D).

## Discussion

Our data show the novel finding that VEGFR-3 signaling regulates absorption and trafficking of dietary lipids from the enterocytes to the circulation. VEGFR-3 is initially expressed in all embryonic endothelia, but becomes highly restricted to the lymphatic endothelium in adult tissues (Kaipainen et al., 1995). However, VEGFR-3 can be found at sites of inflammation and tumor in non-endothelial bone marrow-derived cells (Skobe et al., 2001), stromal dendritic cells (Hamrah et al., 2004) and some VEGFR-3 expression has been described in angiogenic blood endothelial cells (Valtola et al., 1999). The Chy mutant mouse model carries an inactivating mutation in the tyrosine catalytic domain of VEGFR-3. We show that Chy mice have markedly decreased postprandial levels of TGs in the circulation, as compared with controls. Since lipoprotein lipase (LPL) activity was inhibited as previously reported (Otway and Robinson, 1967), the decrease in postprandial TG levels mainly reflects input from the small intestine. Surprisingly, cholesterol levels were also significantly decreased in plasma of Chy mice compared with littermate controls (Figure 1). The significance of this change is unclear and might reflect abnormality of systemic cholesterol metabolism. The lymphatic vasculature regulates removal of cholesterol from peripheral tissues (Lim et al., 2013; Randolph and Miller, 2014) and defective lymphatics impair reverse cholesterol transport (Martel et al., 2013).

Distribution of TGs in different sections of the intestine was strikingly altered in the Chy mouse. Under unchallenged 12-h fasting conditions, there was significant accumulation of TGs in the middle and distal sections of the Chy mouse as compared with control group (Figure 2). Following intragastric administration of triolein, intestines of control mice showed TGs accumulation in the proximal small intestine at 120 min post triolein gavage, about doubled those in fasting. However, in the Chy mouse, TG accumulation (∼threefold compared with fasting levels) was observed throughout the length of the small intestine (Figure 2). This was consistent with impaired proximal TG absorption resulting in more TGs reaching the middle and distal sections. In line with this, enterocytes contained larger cytoplasmic lipid droplets, visualized by Perilipin 3 staining supporting a defect in transfer of the TGs from the enterocyte to the lymphatic system and resulting in increased lipid (TGs and FFAs) excretion in the feces of Chy mice as compared with controls (Figure 2C). VEGF-C in the gut is manly produced by smooth muscle cell and muscle fibers surrounding the lacteals (Nurmi et al., 2015). Postnatal deletion of *Vegfc* causes atrophy of the lacteal, impairs lipid absorption and associates with increased lipid excretion in the feces (Nurmi et al., 2015). In the case of the Chy mouse, the intestinal lymphatics are enlarged (Karkkainen et al., 2004) and our data suggest that they are not functioning in TG transport. Thus, VEGFR-3 signaling in the small intestine appears important during lipid absorption. Individuals with hereditary or primary lymphedema (Milroy’s disease) due to an inactivating mutation in VEGFR-3 kinase domain (Irrthum et al., 2000) often present steatorrhea, which reflects the failure of intestinal lymphatics to adequately transport chylomicrons, with chylous ascites and lymphopenia due to loss of lymph retention (Lee and Young, 1953; Gerstein et al., 1957; Alexander et al., 2010). Retention of chylomicrons in the enterocytes is reported in individuals carrying mutations in the *SAR1B* gene that encodes for sar1b, a GTP-binding protein important for chylomicron trafficking in the enterocytes (Cifarelli and Abumrad, 2018); however, the lymphatic function in these individuals is not affected. VEGFR-3 is highly expressed in LECs and upon binding VEGF-C it dimerizes and undergoes autophosphorylation leading to activation of ERK1/2. It also heterodimerizes with VEGFR-2 leading to activation of AKT (Pajusola et al., 1994; Dixelius et al., 2003; Nilsson et al., 2010). Although VEGF-C can still signal via VEGFR-2 it is possible that the magnitude of VEGF-C/VEGFR-2 signaling might be altered by absence of a functional VEGFR-3. Zhang et al. (2018) have elegantly shown that chylomicron uptake by the lacteals is regulated by dynamic changes of cell-cell junctions via VEGF-A signaling through VEGFR-2. Zippering of lacteal junctions prevents chylomicron uptake and protects mice from diet-induced obesity (Zhang et al., 2018). It is possible that inhibition of VEGFR-3 signaling in the Chy mouse increases VEGFR-2 signaling leading to inhibition of TG uptake by lacteals.

The retention of TGs in enterocytes of the Chy mouse suggests that VEGFR-3 signaling might be involved in promoting efficient TG transfer from enterocytes to lacteals. This is further suggested by our data showing reduced nitric oxide generation in the Chy mouse (Figure 2D). Nitric oxide regulates mobilization of postprandial chylomicrons from the enterocytes into the bloodstream (Hsieh et al., 2015). Disruption of this pathway could be responsible for the accumulation of TGs observed in the enterocytes and mucosa and the subsequent lipid excretion into the feces. Furthermore, VEGF-C binding to VEGFR-3 selectively phosphorylates endothelial nitric oxide synthase (eNOS) at Ser 1177 via the PI3K/Akt pathway in LECs and this effect is not mediated via VEGFR-1 and/or VEGFR-2 (Coso et al., 2012). We hypothesized that generation of nitric oxide followed by VEGF-C binding to VEGFR-3 might affect lacteal permeability, favoring mobilization of TGs from the enterocytes into the lacteals, as it has been proposed (Hsieh et al., 2015). In summary, our study show the novel finding that VEGFR-3 signaling is an important regulator of dietary TG absorption and retention in the enterocytes possibly via generation of NO.

## Author Contributions

TS, NW, and VC ran the experiments and collected and analyzed the data. VC designed the work and wrote the manuscript. All authors critically revised and approved the manuscript.

## Conflict of Interest Statement

The authors declare that the research was conducted in the absence of any commercial or financial relationships that could be construed as a potential conflict of interest.
